# The TGF-β Family: Signaling Pathways, Developmental Roles, and Tumor Suppressor Activities

**DOI:** 10.1100/tsw.2002.178

**Published:** 2002-04-06

**Authors:** Aaron N. Johnson, Stuart J. Newfeld

**Affiliations:** Department of Biology, Arizona State University, Tempe, AZ 85287-1501, USA; Graduate Program in Molecular and Cellular Biology, Arizona State University, Tempe, AZ 85287-1501, USA

**Keywords:** Transforming Growth Factor-β (TGF- β), Activin, nodal, decapentaplegic (dpp), Bone Morphogenetic Protein (BMP), Growth and Differentiation Factor (GDF), serine—threonine kinase receptors, Smad family, pattern formation, organogenesis, limb development, cell cycle, breast cancer, colon cancer, pancreatic cancer

## Abstract

Intercellular communication is a critical process for all multicellular organisms, and communication among cells is required for proper embryonic development and adult physiology. Members of the Transforming Growth Factor-β (TGF-β) family of secreted proteins communicate information between cells via a complex signaling pathway, and family members are capable of inducing a wide range of cellular responses. The purpose of this review is to provide the reader with a broad introduction to our current understanding of three aspects of the TGF-β family. These are the molecular mechanisms utilized by TGF-β signaling pathways, the developmental roles played by TGF-β family members in a variety of species, and the growing list of cancers in which various TGF-β signaling pathways display tumor suppressor activity.

### INTRODUCTION

Over the last decade and a half, Transforming Growth Factor-β (TGF-β) signaling pathways have been widely examined, and they have been shown to be essential for embryonic patterning, organogenesis, and adult tissue homeostasis[1,2,3]. Phylogenetic studies and interspecific experiments have identified homologous TGF-β family members in humans and all animal model organisms[4,5,6]. The goal of this review is to provide a broad introduction to the field of TGF-β signaling and to present recent advances in our understanding of this highly influential pathway. We discuss biochemical, embryological, and genetic studies in vertebrates and invertebrates that identify major features of TGF-β signaling.

### TGF-β FAMILY

Molecular phylogenetic analyses suggest that the TGF-β family originated roughly 1.3 billion years ago, prior to the divergence of nematodes from the common ancestor of arthropods and vertebrates[5]. For perspective, the TGF-β family does not predate the divergence of plants, animals, and fungi (dated at roughly 1.6 billion years ago)[7], suggesting that TGF-β family members are found only in animals. In light of this long evolutionary history, the basic features of TGF-β signaling pathways (Fig. 1A) have been impressively conserved.

#### Subfamilies and Interspecific Conservation

Sequence analyses of the TGF-β family have identified significant amino acid conservation across species (Fig. 1B). Two major TGF-β subfamilies have been identified based upon amino acid identities. The largest and most widely distributed subfamily is the Dpp/BMP subfamily (Decapentaplegic/Bone Morphogenetic Protein). This subfamily has members in flies, nematodes, and vertebrates (reviewed in [8]). The level of sequence similarity between Dpp/BMP subfamily members in distant species is striking. For example, a comparison of Dpp from flies with human BMP2 and BMP4 revealed >75% amino acid identity[9]. The wide species distribution of the Dpp/BMP subfamily suggests that it is the oldest TGF-β subfamily. The other major TGF-β subfamily is the TGF-β/Activin subfamily. To date, no nematode TGF-β family members have been assigned to the TGF-β/Activin subfamily with statistical confidence. The absence of obvious TGF-β/Activin subfamily members in nematodes suggests that this subfamily arose after the separation of nematodes from the common ancestor of arthropods and vertebrates (roughly 950 million years ago)[7]. Sequence similarities between TGF-β/Activin subfamily members are not quite as high as in the Dpp/BMP subfamily. A comparison of Activin from flies and mammals reveals only 45% amino acid identity[5].

This extraordinary level of sequence conservation in the Dpp/BMP subfamily is reflected in the ability of subfamily members to function correctly in interspecific experiments. For example, when human BMP2 or BMP4 is expressed in *Drosophila*, it is able to rescue *dpp* mutant phenotypes[4]. Conversely, when mammalian osteoblast cell cultures are incubated with Dpp protein, they are able to differentiate into mature bone tissue[10]. These experiments suggest that closely related family members have the ability to perform the functions of their homologs when expressed in distant species. As described below, interspecific experiments with TGF-β receptors and signal transducers have demonstrated that these downstream components of TGF-β signaling pathways are also functionally conserved (Fig. 1C). The interspecific functionality of TGF-β ligands, receptors, and signal transducers has been exploited in a variety of ways to improve our knowledge of this signaling pathway.

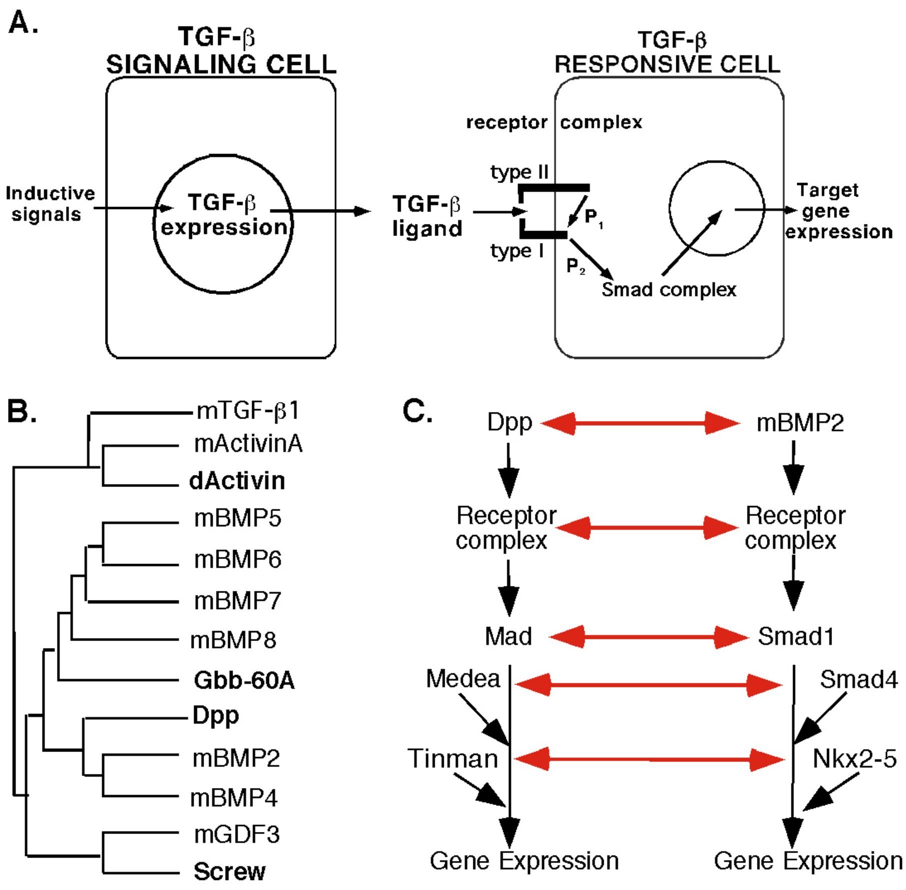

**FIGURE 1.** The TGF-β signaling pathway, family members, and cross-species functionality. (A) The TGF-β signaling pathway. Inductive signals may be either TGF-β family or non-TGF-β molecules. $P_1$ indicates the phosphorylation of the Type I receptor by the Type II receptor in response to ligand binding. $P_2$ indicates the phosphorylation of Smad signal transducers by the Type I receptor in response to its phosphorylation. Phosphorylation induces a complex of cytoplasmic Smad proteins to translocate to the nucleus. Transcriptional changes in the responsive cell include gene activation and often further TGF-β signaling. (B) TGF-β family members. Relationships based upon amino acid similarity in the ligand region are shown for a representative set of mammalian and fly TGF-β family members. There are two major subfamilies in the TGF-β family, the TGF-β/Activin subfamily (top three sequences) and the Dpp/BMP subfamily (remaining sequences). Each *Drosophila* family member is more closely related to mammalian family members than to other *Drosophila* family members. (C) Cross-species functionality. Components of the Dpp (*Drosophila*) and BMP2 (mammal) signal transduction pathways are shown. Downward arrows indicate the movement of inductive signals and the point at which each component participates in signal transduction. Each pathway component in *Drosophila* is most closely related to the indicated component from mammals. Horizontal arrows indicate that cross-species experiments have been performed in both directions. In each case, the *Drosophila* protein functioned properly in mammalian cells and the mammalian protein functioned properly in *Drosophila* embryos.

### SIGNALING PATHWAYS

We have divided the process of TGF-β signaling into roughly four steps. First, a TGF-β ligand is transcribed, translated, and secreted from a signaling cell. Second, the ligand traverses the intercellular space and interacts with receptors on the surface of a receiving cell. Third, these receptors activate ligand-specific intracellular messenger proteins known as Smads. Fourth, activated Smads translocate to the nucleus and influence target gene expression.

As with many other signal transduction pathways, each step of the TGF-β pathway is under strict regulation. For example, the regulation of ligand transcription and secretion influences how positional information is disseminated during development both spatially and temporally. Furthermore, the manner in which positional information is interpreted in receiving cells involves the regulation of the signal transduction apparatus. Thus, pathway regulation is a significant part of the mechanics of TGF-β signal transduction.

### Transcriptional Regulation

Transcriptional regulation of TGF-β family members is complex due to the influence of the large number of distinct regimes that are utilized. Some of the molecular mechanisms involved in regulating the transcription of the Dpp/BMP subfamily member *dpp* during development in *Drosophila* have been brought to light. Three regions, each containing distinct cis-regulatory sequences, have been identified at the *dpp* locus. Reporter gene studies utilizing enhancer elements from each of these regions[11,12,13] have shown that discrete cis-regulatory elements are sufficient to direct *dpp* expression in subsets of its normal expression pattern. Reporter gene studies in mice of two TGF-β family members, BMP2 (a *dpp* homologue) and Nodal, have given similar results[14,15]. These experiments have led to a developmental model in which spatially and temporally dynamic patterns of expression of TGF-β family members are induced through the actions of separate enhancer elements.

The transcriptional activity of enhancers that regulate TGF-β expression are influenced by TGF-β and non-TGF-β signaling pathways. Many TGF-β proteins participate in positive transcriptional autoregulation, including TGF-β1, -β2, and -β3[16,17], nodal[18], and Dpp/BMP subfamily members[19,20]. For example, during *Drosophila* development, *dpp* expression in the visceral mesoderm is initiated by the homeotic gene Ultrabithorax via well-characterized enhancer elements. Dpp expression is then required to maintain Ultrabithorax expression. Continuous Ultrabithorax expression in turn maintains *dpp* expression[21,22]. Thus, *dpp* expression is indirectly autoregulatory via Ultrabithorax. BMP2 may also be autoregulatory. In experiments using osteoblast cell lines, incubation with BMP2 protein activated a BMP2 reporter construct[15].

The expression of TGF-β family members is also controlled by other signaling pathways, including those of the Epidermal Growth Factor Receptor, Wingless, and Hedgehog[23,24,25,26]. One example of positive regulation is seen following epithelial to mesenchymal transition in mammalian cell culture. In this system, sustained Raf activity (Raf is a member of the Epidermal Growth Factor Receptor signal cascade) induces TGF-β1 production[25]. A second example is seen in human lung epithelial cells. Here the Epidermal Growth Factor Receptor responsive transcription factor complex AP-1 (Jun/Fos) initiates transcription of TGF-β1 and then mediates TGF-β autoregulation in a mechanism similar to that described for Dpp and Ultrabithorax[17]. Alternatively, the Wingless pathway can repress *dpp* in *Drosophila*. One example of negative regulation is seen in *Drosophila* leg imaginal disks. In this tissue, *wingless* and *dpp* show mutually exclusive expression patterns suggesting mutual antagonism at the transcriptional level[26,27]. A second example is seen in the visceral mesoderm. In this tissue, dTCF (a downstream effector of Wingless signaling) represses the expression of a *dpp* reporter gene in the visceral mesoderm[28]. Interestingly, the expression of this *dpp* reporter construct is activated by Wingless signaling in anterior regions of the visceral mesoderm and repressed by Wingless signaling in more posterior regions. These results suggest that *dpp* activation or suppression in the visceral mesoderm is not simply due to the presence or absence of a Wingless signal but may be regulated by the level of Wingless signaling[28].

### Processing and Activation

Once transcribed and translated, TGF-β proteins undergo multiple post-translational modifications (Fig. 2). The first is cleavage of the proprotein[29,30]. Subsequent to cleavage, the

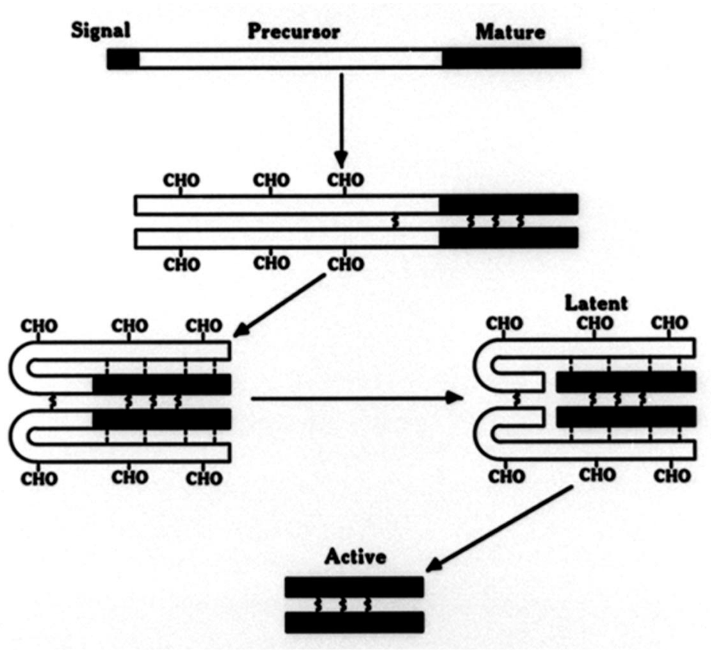

**FIGURE 2**. TGF-β ligand processing and activation. Schematic representation of the steps involved in the processing and activation of a typical TGF-β protein. Prior to secretion, TGF-β family members associate into disulfide-bonded dimers. These can be homo- or heterodimers, depending upon the family member. Protein folding then results in the formation of noncovalent bonds within each monomer of the dimer. After proteolytic cleavage of the C-terminal ligand domain from the N-terminal portion of the protein, the disulfide and noncovalent bonds act to maintain the molecule in a fully folded, latent form. The protein is secreted in this form, but it is incapable of binding to receptors until the N-terminal region is dissociated from the ligand region.

carboxy terminus of the full-length protein will become the active TGF-β ligand while the amino terminus, often termed the latency-associated peptide (LAP), remains noncovalently associated with the carboxy terminus even after secretion[31]. The TGF-β ligand is secreted as a latent dimer from the signaling cell[32]. Cotransfection experiments with LAP and mature TGF-β protein show that intracellular ligand processing and dimerization require the presence of LAP[33]. Thus TGF-β family members are secreted as latent cytokines, with latency being conferred by LAP.

Prior to secretion, TGF-β ligands also form heteromeric, covalent complexes with Latent TGF-β Binding Protein (LTBP)[34,35]. LTBP expression is upregulated coincidentally with TGF-β expression, suggesting that these proteins are coregulated[36]. LTBP–ligand association is specific to TGF-β subfamily members. The LTBP acts to regulate TGF-β ligand activity by perpetuating latency during intercellular translocation while simultaneously targeting the ligand to the appropriate cells[37]. An analysis in bovine endothelial cells revealed that LTBP-bound TGF-β interacts with the transmembrane insulin-like growth factor receptor 2 (IGFR2). This interaction increases the concentration of TGF-β at the cell surface. Antibody inhibition of the TGF-β/LTBP complex interaction with IGFR2 results in a decrease of available TGF-β[38]. Further experiments demonstrate that LTBP cross-links to the extracellular matrix via a transglutaminase-dependent mechanism[39]. Interestingly, TGF-β1 signaling has been shown to negatively regulate transglutaminase transcription in mink lung epithelial cells[40], suggesting the possibility of ligand autoregulation in the intercellular space. These studies indicate that LTBP acts not only as a TGF-β targeting protein but also as a TGF-β storage mechanism, thus facilitating ligand accumulation in the extracellular matrix until activation. To date, LTBP has been exclusively characterized in vertebrate systems. The identification of invertebrate LTBP homologs may reveal additional evolutionarily conserved aspects of TGF-β signaling pathways.

Activating TGF-β isoforms involves the removal of latency-conferring proteins. *In vitro* studies have implicated a number of proteins and nonenzymatic treatments that activate TGF-β proteins, including plasmin[41], calpain[42], and extreme pH. Subsequent *in vivo* studies have produced a model in which the secreted TGF-β–LAP complex bound to LTBP associates with components of the extracellular matrix that stimulate the release of the TGF-β–LAP complex. The TGF-β–LAP complex then translocates to the cell membrane where it is a substrate for membrane-bound proteins. Integrin receptors αVβ1, 3, and 6 have been identified as TGF-β–LAP binding proteins[43] that release active TGF-β through a plasmin-dependent mechanism. Recent work has also implicated thrombospondin-1 as an important ligand coactivator with plasmin[44,45]. Thus, activation of TGF-β subfamily members involves many layers of regulation.

### Extracellular Regulation

Although the mechanisms of activation for Dpp/BMP subfamily members remain to be characterized, several regulatory mechanisms affecting ligands of this subfamily have been identified. Extensive extracellular ligand regulation occurs through the activity of ligand-specific antagonists. Short gastrulation (Sog) in *Drosophila* and its vertebrate homolog Chordin specifically bind and antagonize extracellular Dpp/BMPs[46,47,48,49]. Recently, *Xenopus* Twisted-gastrulation (Tsg) has been shown to synergize with Chordin to antagonize BMP2 and BMP4[50]. Noggin and the DAN family of proteins are likewise BMP-specific antagonists in vertebrates[51]. An additional level of regulation is provided by antagonists of TGF-β antagonists such as Tolloid in *Drosophila* and Xolloid in *Xenopus*. Xolloid is a Chordin-specific antagonist that binds and cleaves BMP–Chordin complexes, releasing BMP to continue its translocation to the receiving cell surface[52,53]. Mouse BMP1, human Tolloid1, and *Drosophila* Tolloid function similarly to Xolloid, indicating the conservation of extracellular antagonism and release mechanisms.

Transient ligand antagonists play a role in many developmental systems by creating spatial differences in the concentration of active ligands, often termed morphogen gradients. Long-range gradients are required to deliver the correct positional information to cells throughout a tissue or organ and are widely utilized for pattern formation during development in many organisms. The formation of Dpp/BMP subfamily ligand activity gradients had been proposed to be under the sole control of extracellular regulation. Under this model, ligands rapidly diffuse from a signaling source, while the activity gradient is established through extracellular interactions with Sog/Chordin and Tolloid[48]. The ligand gradient is then superimposed on spatial differences in receptor expression to generate an overall gradient of positional information[54].

Recently, studies of extracellular *dpp* trafficking have indicated that endocytosis and intracellular ligand regulation are also involved in gradient formation in *Drosophila*[55]. This idea derived from studies of mutant tissues in otherwise normal individuals. These studies in *Drosophila* show that the loss of specific endocytic processes prevents the spread of a fluorescently labeled Dpp molecule. Furthermore, by promoting the degradation of all endocytosed proteins in these individuals, the range of Dpp diffusion was further decreased[56]. These results strongly argue that, in some instances, the Dpp ligand is endocytosed and trafficked intracellularly as part of the establishment of a morphogen gradient.

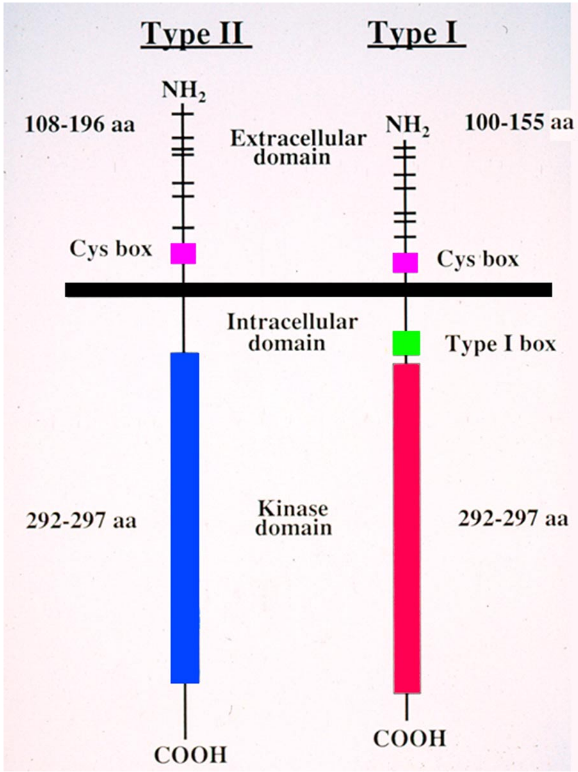

**FIGURE 3.** TGF-β receptor structure. Schematic representation of the Type I and Type II receptors. Major features such as the cysteine box, the kinase domain, and the Type I or GS box are shown. The size range of the extracellular and intracellular domains, in numbers of amino acids, is shown. Perpendicular lines represent additional cysteines in the extracellular domain.

### Signal Transduction

#### *Type I and Type II Transmembrane Receptors*

The Type I and Type II receptors for TGF-β family ligands are transmembrane receptor serine/threonine kinases (Fig. 3). A molecular phylogenetic analysis showed that Type I and Type II receptors are distantly related to each other and suggests that Type I receptors diverged from an ancestral Type II receptor[5]. Within each receptor's family — Type I (Fig. 4A) and Type II (Fig. 4B) — significant amino acid conservation is seen across species. The functional conservation of TGF-β receptors is impressive. For example, a complex containing a Type I receptor from *D. melanogaster* (Saxophone) and a Type II receptor from *Caenorhabditis elegans* (daf-4) binds human BMP2 with high affinity[57].

The association of an active TGF-β ligand dimer with a Type II transmembrane receptor serine/threonine kinase initiates signal transduction. Type II receptors are thought to be constitutively active kinases but require ligand association to functionally interact with Type I receptors. Upon ligand binding, a heteromeric receptor complex forms. Initially, this receptor complex was thought to be composed of a single pair of Type I and Type II receptors per ligand dimer[58,59]. Subsequently, two-dimensional gel analysis and receptor precipitation experiments revealed that receptor complexes can contain multiple Type I and multiple Type II receptors[60]. Upon complex formation, the Type II receptor phosphorylates the GS region of the Type I receptor, a highly conserved domain bearing the amino acid sequence SGSGSG. This activates the serine/threonine kinase activity of Type I receptors. Activated Type I receptors are then able

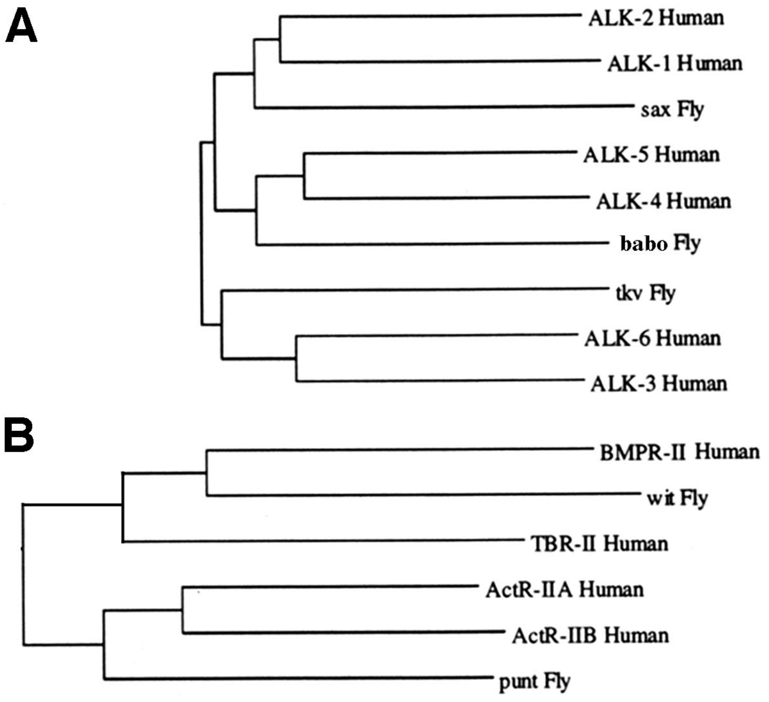

**FIGURE 4.** TGF-β receptor families. Relationships based upon amino acid similarity are shown for a representative set of human and fly TGF-β receptors. (A) Type I receptors. (B) Type II receptors. The Type I and Type II receptor families are distantly related to each other. As in the TGF-β family, each *Drosophila* receptor is more closely related to a human receptor than to another fly receptor. For each fly receptor, there are two closely related human receptors. In all cases, except *wit*, the relationship between the human and fly receptors in a cluster matches the relationship between the species. This suggests that numerous gene duplication events occurred in the mammalian lineage after its divergence from the arthropod lineage. Interestingly, homologous human and fly receptors do not always function in the pathway of homologous TGF-β family members. For example, Saxophone signals for DPP/BMP subfamily members, while its homologs, ALK-1 and ALK-2, signal for TGF-β/Activin subfamily members.

to phosphorylate the conserved SSXS motif of receptor-regulated Smads (R-Smads; see below), the intracellular signal transducers. Due to their critical role in R-Smad phosphorylation, Type I receptors serve as the center point for intracellular signaling.

The specificity of ligand–receptor recognition has been a difficult issue in the field of TGF-β signaling, and numerous inconsistencies exist between studies of ligand–receptor interactions. For example, genetic analyses have shown that the *Drosophila* Type II receptor Punt signals for the Dpp/BMP subfamily member Dpp[61]. However, biochemical analyses demonstrated that Punt binds the TGF-β/Activin subfamily member Activin with high affinity[62]. A second example derives from studies of the *Drosophila* Type I receptors Thickveins and Saxophone. Genetic analysis of mutant cells in otherwise wild-type individuals suggested that both Thickveins and Saxophone transduce Dpp signals in the developing wing[63]. However, experiments in embryos with injected Thickveins and Saxophone mRNAs showed different results. One experiment showed that a constitutively active form of Thickveins could induce Dpp-dependent cell types in a *dpp*-deficient embryo but that a constitutively active form of Saxophone could not[64]. A second experiment showed that injection of a dominant-negative form of Saxophone did not block the effects of ectopic Dpp expression but that a dominant-negative form of Thickveins could[65]. Thus, in contrast to the genetic analyses, the injection experiments suggest that Dpp only signals through Thickveins. It appears that confidence in assessments of ligand–receptor specificity can only be derived from the convergence of studies employing a variety of methods.

### Receptor Regulation

TGF-β receptor transcription is often regulated by TGF-β signals. In most instances TGF-β signaling downregulates Type I and Type II receptor expression[66]. Alternatively, there are developmental contexts where TGF-β signaling promotes receptor expression. For example, BMP2 signaling in ventral midbrain stem cells induces the expression of the Type I receptor BMPR-1B presumably by signaling through BMPR-1A[67]. This activation of BMPR-1B is necessary for proper CNS differentiation and patterning in mice. The transcription of TGF-β receptors is also influenced by other non-TGF-β signals. For example, in wing development the Dpp Type I receptor Thickveins is repressed by the DNA-binding protein Master of thickveins[68]. *Master of thickveins* is transcribed, like *dpp*, in response to Hedgehog signals. Thus cells that express *dpp* and *Master of thickveins* are unable to respond to Dpp signaling. To date, no vertebrate homolog of *Master of thickveins* has been characterized.

In addition to transcriptional regulation, the function of TGF-β receptors can be modulated by protein–protein interactions. For instance, FKBP12 inhibits Type I receptor activation[69] by binding to the GS domain and capping the receptor's phosphorylation site[70]. A second receptor inhibitor, BAMBI (BMP and Activin membrane-bound inhibitor), is a TGF-β Type I pseudoreceptor that inhibits TGF-β, Activin, and BMP signaling in *Xenopus*. BAMBI lacks an intracellular kinase yet maintains the ability to associate with other TGF-β receptors[71]. Thus, BAMBI effectively ties up TGF-β family ligands and receptors at the cell surface and prevents the activation of Smad proteins. So far, BAMBI proteins have only been identified in vertebrates[72,73].

Betaglycans, first identified as TGF-β Type III receptors, also participate in protein-mediated receptor regulation. Originally, Type III receptors were thought to present TGF-β/Activin subfamily ligands to their Type II receptors, thereby facilitating the formation of ligand–receptor complexes[74]. This proposal was derived from cell culture experiments in which betaglycan increased the affinity of TGF-β1 and TGF-β2 for their receptors[75,76]. Alternatively, betaglycans can act as TGF-β/Activin subfamily antagonists. One example of betaglycan antagonism involves Activin and Inhibin. Inhibin competes with Activin for specific Type II receptors. Activin-bound Type II receptor can bind the cognate Type I receptor and stimulate it to transmit signals, while an Inhibin-bound Type II receptor cannot. In *in vitro* studies, betaglycan/Inhibin complexes have a higher affinity for the Type II receptor than does Inhibin alone[77], suggesting that in the presence of betaglycan, Inhibin can outcompete Activin, thus attenuating Activin signaling. Recently, betaglycan antagonism was demonstrated *in vivo* in mammalian renal epithelial cells[78].

### Smad Signal Transducers

Smad proteins transduce the TGF-β signal from activated receptor complexes to the nucleus via a phosphorylation-dependent mechanism. A variety of intracellular proteins, including antagonists, coactivators, corepressors, and transcription factors, interact with Smads during their translocation from the cell membrane to the nucleus, thus influencing the fate of the TGF-β signal within the cell. Ultimately these multiprotein interactions provide the specificity by which the TGF-β signal alters gene expression within the nucleus of the receiving cell.

Smads were initially identified in screens for second-site mutations that enhanced the severity of mutations in TGF-β family members. The first Smad to be characterized was *Mothers against dpp* (*Mad*) in *Drosophila*. *Mad* mutations were identified by their ability to act as

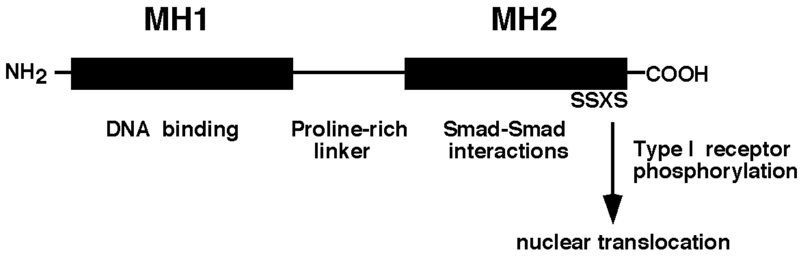

**FIGURE 5**. Smad protein structure. Smad family members typically contain conserved Mad-Homology (MH) domains separated by a divergent proline-rich linker region. The MH1 domain appears to be responsible for transcriptional activation, while the MH2 domain appears necessary for forming multi-Smad complexes. Type I receptors phosphorylate the SSXS motif in the MH2 domain of pathway-specific Smads (R-Smads). This leads to the recruitment of a multipathway Smad (Co-Smad) to form a heteromeric complex. The multimeric complex then translocates to the nucleus and participates in the transcriptional activation of target genes.

dominant maternal enhancers of *dpp* mutant phenotypes[79,80]. Subsequently *sma-2*, *sma-3*, and *sma-4* were identified in *C. elegans* based on their ability to phenocopy some *daf-4* (TGF-β Type II receptor) mutant phenotypes[81]. Sequence comparison of the *sma* genes with *Mad* shows extensive amino acid conservation. The conserved sequences were used to identify similar proteins in vertebrates. The resulting multigene family is known as the Smads, a name incorporating *sma* and *Mad*[82].

Structurally, Smad proteins contain two functionally conserved Mad Homology (MH) domains (Fig. 5). The MH1 domain, located near the N-terminus of the protein, is known to bind DNA. The MH1 domain is also involved in determining transcription factor associations. For example, Smads lacking the MH1 domain showed a loss of cofactor specificity in *Xenopus*[83,84]. The MH2 domain, located at the C-terminus, participates in protein–protein interactions and houses the well-characterized SSXS phosphorylation site[85]. The MH2 domain is involved in establishing both receptor–Smad and Smad–Smad interactions[86].

Individual Smads from different vertebrate species show very high levels of amino acid identity. For example, *Xenopus* and human Smad1 are 95% identical. Of the vertebrate Smads, Smad1 shows the most sequence similarity to *Mad* (76% amino acid identity with *Xenopus* Smad1)[5]. This high level of conservation is reflected in the ability of Smads to function correctly (performing the function of their homolog) in distant species. For example, in *Xenopus*, *Drosophila* Mad mimicked Smad1 in mesoderm induction assays[87]. In two examples from *Drosophila*, injected human Smad4 rescued *Medea* mutant phenotypes[88], and a transgenic analysis showed that closely related human and *Drosophila* Smads generate the same phenotypes when overexpressed[6].

To date, three types of Smad proteins have been identified (Fig. 6), including receptor-regulated Smads (R-Smads), common mediator Smads (Co-Smads), and inhibitory Smads (I-Smads). R-Smads, such as *Mad* in *Drosophila*[87], are direct targets of Type I receptor phosphorylation[89,90]. R-Smads are phosphorylated only by the receptors for a single TGF-β subfamily. For example, in vertebrates Smad1, Smad5, and Smad8 are downstream of Dpp/BMP receptor signals, while Smad2 and Smad3 are downstream of TGF-β/Activin subfamily receptors[91]. Interactions between Smad2 and Smad3 and their Type I receptors are mediated by SARA (Smad anchor for receptor activation) proteins in humans and in *Xenopus*[92]. To date, no counterpart for SARA has been identified for Dpp/BMP signaling Smads.

Co-Smads, including *Drosophila* Medea and vertebrate Smad 4, act in association with R-Smads from multiple pathways. Co-Smads lack the SSXS phosphorylation sequence in their MH2 domain, making them unresponsive to direct Type I receptor activation[93]. However,

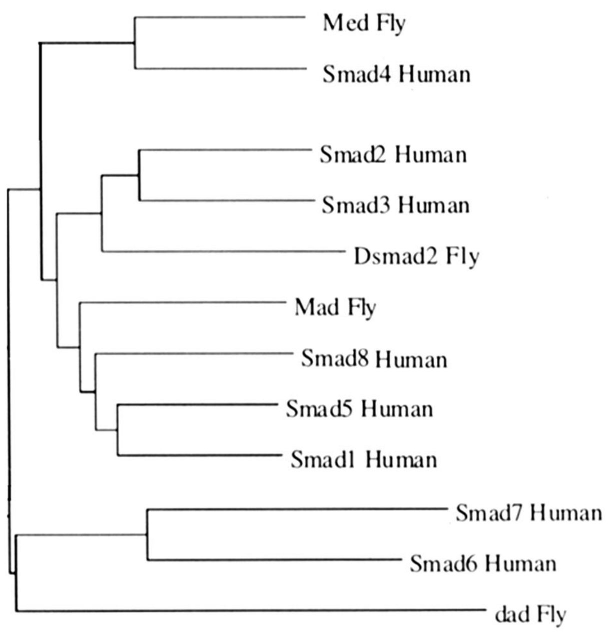

**FIGURE 6.** Smad family. Relationships based upon amino acid similarity are shown for a representative set of human and fly Smad proteins. As in the TGF-β family and the receptor families, each *Drosophila* Smad is more closely related to a human Smad than to another fly Smad. Three major subgroups can be identified. One cluster consists of Med and hSmad4 (Co-Smads). These Smads can transduce signals from both TGF-β subfamilies. A second cluster consists of two subgroups. All members of the cluster are pathway-restricted signal transducers (R-Smads). Mad, hSmad1, hSmad5, and hSmad8 are dedicated to the DPP/BMP subfamily. dSmad2, hSmad2, and hSmad3 are dedicated to the TGF-β/Activin subfamily. A third cluster consists of Dad, hSmad6, and hSmad7. These Smads antagonize signals of both TGF-β subfamilies (I-Smads).

R-Smads and Co-Smads form multimeric protein complexes with each other[93,94]. In cell culture, R-Smad activation is necessary for Co-Smad accumulation within the nucleus[93,95]. Enhancer regions containing R-Smad binding sites also house Co-Smad binding sites[96]. These data suggest that multimeric protein complex formation is required for Co-Smads to exert an effect on target genes, while R-Smads have the ability to function in the nucleus with or without Co-Smads.

Two recent studies suggest that R-Smads and Co-Smads influence target gene expression as intermediaries rather than as direct transcriptional activators. Specifically, studies in mammalian cell culture showed that Smad2 and Smad3 can stimulate the p38 and the Ras/Raf mitogen-activated protein kinase (MAPK) signaling pathways, respectively[97,98]. In these studies, TGF-β signals induced the transcription of target genes that have no known Smad2 or Smad3 regulatory sequences. Importantly, removing Smad2 or Smad3 activity in these assays abolished target gene expression, indicating that these Smads are necessary for TGF-β–mediated activation of MAPK pathway target genes.

I-Smads, the third class of Smads, are structurally distinct from other Smads. Specifically, I-Smads have a highly divergent MH1 domain[5] and their MH2 domain lacks the SSXS phosphorylation site[99]. I-Smads, such as *Daughters against dpp* (*Dad*) in *Drosophila*[99] and Smad6 and Smad7 in vertebrates[100,101], are thought to compete with R-Smads for Type I receptor binding sites, thus preventing R-Smad activation. For example, ectopic Dad expression in developing *Drosophila* wings produces a phenotype similar to that observed in *dpp*-deficient wings[99]. Interestingly, enhancer trap studies have shown that Dpp signaling is required for Dad expression[99]. Thus, I-Smads may complete a negative feedback loop temporally limiting the transduction of TGF-β signals.

#### Smad Regulation

Recent investigations have identified a number of coactivators, corepressors, transcription factors, and antagonists functioning to modify Smad activity in TGF-β signal transduction. For example, Smads interact with histone acetyl transferases (HATs) and histone deacetylases (HDACs) to remodel chromatin structure. Smad2, Smad3, and Smad4 are known to interact with the HAT domain of the mammalian transcriptional coactivator p300/CBP in a ligand-dependent manner[102,103]. In *Drosophila*, mutations in *nejire*, the p300/CBP homolog, reduce *dpp* target gene expression[104]. These studies suggest that HATs act to accentuate TGF-β signaling. HDACs act to reverse the effects of HATs. The homeodomain protein TGIF[105,106], the oncogene *c-ski*, and the related protein SnoN bind active Smad2/Smad4 and Smad3/Smad4 complexes and then recruit HDACs to the Smad complex[107,108]. Once associated with the Smad complex, HDACs act to repress TGF-β–mediated gene expression[107,108]. The recruitment of HATs and HDACs to Smad complexes modifies the ability of TGF-β signaling to regulate target gene expression.

Smad activity is also modulated via interaction with ubiquitin E3 ligases. These ligases target Smads for ubiquitination and subsequent proteosome degradation[109,110,111]. Specifically, the E3 ligases Smurf1, Smurf2, and Roc1-SCF stimulate the ubiquitination of R-Smads in mammalian cell culture and in *Xenopus* assays.

The specificity of transcriptional regulation by Smads is dependent upon interactions with transcription factors. For example, Smads cooperate with the transcriptional activator FAST-1 (Forkhead Activin Signal Transducer-1) to transcribe Activin-inducible genes in *Xenopus* and with FAST-2 to transcribe Activin-inducible genes in mice[18,96,112]. Alternatively, in *Drosophila*, Mad competes with the transcriptional repressor Brinker to activate *zerknullt* transcription[113,114].

#### Target Genes

We have divided the transcriptional targets of TGF-β signaling into three general categories: TGF-β pathway components (discussed above), cellular identity genes during development (Table 1), and cell cycle control genes in adult physiology (Table 2). The regulation of receptor expression is an example of a target in the first category. The regulation of Hox gene expression is an example of a target in the second category. The regulation of cyclin-dependent kinase inhibitor expression is an example of a target in the third category.

### DEVELOPMENTAL ROLES

During development, TGF-β signaling frequently acts to promote the differentiation of pluripotent cells into specific cell types. In *Drosophila*, for example, Dpp signaling activates transcription of *tinman* in the dorsal mesoderm. *tinman* expression is required for the formation of mesoderm derivatives such as the dorsal vessel (heart) and the somatic muscles[3]. Interestingly, this role of Dpp/BMP signaling is conserved in vertebrates. The vertebrate *tinman* homolog *Nkx2.5* is responsive to BMP signaling, and *Nkx2.5* expression is necessary for cardiac mesoderm specification[115]. During development in vertebrates, homeobox genes that specify particular cell fates are often targets of Activin signaling. For example, *Mix.2* (a mesendodermal marker in *Xenopus*) and *pitx2* (a transcription factor implicated in left–right asymmetry in mouse) are induced by Activin signaling.

The ability of TGF-β family members to induce tissue-specific genes is utilized many times during animal development. We have divided the known roles of TGF-β signaling during development into three classes: axis specification, gastrulation, and left–right asymmetry in early

**TABLE 1**
**Representative TGF-β Developmental Targets**

| Ligand | Target | Organism | Tissue | Reference |
|---|---|---|---|---|
| TGF-β | Slug | Chick | Endocardium | 169 |
| Activin | Mix.2 | Frog | Primordial mesoderm | 96,235 |
| | GATA5 | Zebrafish | Blastoderm | 145 |
| nodal | Pitx.2 | Frog | Presumptive mesoderm | 236 |
| | goosecoid | Zebrafish | Blastula | 237 |
| BMP2/4 | BMPRI-A | Mouse | Neural crest cells | 67 |
| | Tlx-2 | Mouse | Primitive streak | 123 |
| | Dlx-5 | Mouse | Presumptive osteoblasts | 238 |
| | Nkx2.5 | Chick | Cardiac mesoderm | 115 |
| | Msx1 | Frog | Blastomere | 239 |
| Dpp | tinman | Fly | Dorsal mesoderm | 3 |
| | labial | Fly | Endoderm | 20 |
| | Ubx | Fly | Visceral mesoderm | 19 |
| | optimotor blind | Fly | Wing disk | 240 |
| | spalt | Fly | Wing disk | 241 |
| | dpp | Fly | Visceral mesoderm | 22 |
| | Dad | Fly | Wing disk | 99 |

**TABLE 2**
**Representative TGF-β Cell Cycle Targets**

| Ligand | Target | Organism | Tissue | Reference |
|---|---|---|---|---|
| TGF-β | c-Myc | Cell culture | Mammary epithelial cells | 205 |
| | p21 | Cell culture | Human hepatoma cells | 206 |
| | cdc-25 | Mouse | Liver | 242 |
| BMP2/4 | p21 | Cell culture | Neuroblasts | 211 |
| Dpp | cyclin E | Fly | Eye disk | 215 |

embryos; tissue patterning during middle stages of development; and organ specification during the last stages of development. We refer to studies from several model organisms.

### Axis Formation, Gastrulation, and Left–Right Asymmetry

#### *Mouse — Axis Formation*

During the earliest stages of embryonic development, pluripotent cells need to acquire positional identities along the anterior–posterior, dorsal–ventral, and proximal–distal axes. In vertebrates, positional information associated with left–right asymmetry is also important. Differentiation of cells during mouse development begins during the morula stage, when the embryo consists of roughly 16 cells. The morula is a solid ball of cells, and its outer cells will form the trophectoderm, the tissue responsible for interacting with the uterine wall. The inner cells (the inner cell mass) continue to proliferate as pluripotent cells. The inner cell mass will eventually give rise to two structures: the epiblast and the primitive endoderm.

In mice, the first axis of the embryo proper to form is the proximal–distal axis, with proximal referring to the region nearest to the placenta. A proximal–distal gradient of expression of the TGF-β family member Nodal within the epiblast determines embryonic proximal–distal polarity. The anterior–posterior axis forms next, and Nodal signaling in the epiblast is also important for the specification of anterior and posterior cell fates. Nodal expression from the presumptive posterior region influences anterior cell fates via a canonical TGF-β signaling pathway involving Smad2 and FAST-1. Posterior cell fates are specified by Nodal via an unknown mechanism[116,117,118]. Extraembryonic tissues derived from the trophectoderm also affect epiblast axial patterning. For example, BMP4 is expressed outside the embryo, yet mice mutant for BMP4 and its signal transducer Smad1 show defects in axis formation[119,120]. Roles for TGF-β family members in dorsal–ventral axis formation in mice have not yet been identified.

### Mouse — Gastrulation and Left–Right Asymmetry

Gastrulation in mice occurs with the differentiation and migration of mesodermal cells from the primitive streak. Mice lacking Dpp/BMP subfamily signaling pathway components show defects in mesoderm formation. BMP4 mutant mice fail to express the mesodermal marker (T)Brachyury[121]. Mice lacking BMP Type I receptors also show gastrulation defects[121,122]. *Tlx-2* is likely a BMP target gene in mesoderm induction, and its deletion also results in embryos that fail to form mesoderm[123]. Other TGF-β family members, including Activin, Nodal, and several TGF-β isoforms, have been implicated in gastrulation[116,120,124,125]. Subsequent to gastrulation, left–right asymmetry is established by Nodal signaling[118].

### Chick — Axis Formation and Gastrulation

In the chick, the dorsal–ventral axis forms first in the two-layer blastoderm-stage embryo without input from TGF-β family members. The anterior–posterior axis is specified next, as a dense region of cells, the posterior marginal zone, defines the posterior region of the embryo. At this stage, the embryo is comprised of the hypoblast, from which the extraembryonic tissues are derived, and the epiblast (containing the posterior marginal zone), which is the progenitor of the embryo proper. The posterior marginal zone gives rise to the primitive streak, and gastrulation initiates as cells migrate through the streak. The TGF-β family member cVg1 is normally expressed in the posterior marginal zone, and grafts of cVg1 produce an ectopic primitive streak[126], suggesting that cVg1 is involved in streak formation and gastrulation. Proximal–distal axis formation in chicks is discussed in the section on limb formation.

### Chick — Left–Right Asymmetry

Hensen's node develops at the anterior end of the primitive streak and acts to induce growth of the developing tissue layers. Left–right asymmetry develops as tissues surrounding Hensen's node are exposed to asymmetric gene expression. The expression of several TGF-β family members is associated with Hensen's node, and Activin, Nodal, and BMP4 have been implicated in left–right asymmetry[127,128,129].

### Frog and Fish — Axis Formation

In *Xenopus*, an unfertilized egg is divided into a visibly pigmented animal pole and a yolk-containing vegetal pole. The animal–vegetal axis will form the future anterior–posterior axis, with anterior toward the animal pole. Following fertilization, the *Xenopus* embryo undergoes a cortical rotation such that the point of sperm entry in the animal cap defines the ventral pole while the point opposite in the vegetal region becomes the dorsal pole. Importantly, the dorsal pole of the blastula becomes the site of the Nieuwkoop center, a signaling region that induces dorsal–ventral polarity.

Many maternally contributed mRNAs are present in the *Xenopus* egg, including the TGF-β family members Activin, Vg1, BMP2, BMP4, and BMP7. These ligands play a variety of roles in dorsal–ventral patterning and gastrulation. The Nieuwkoop center is believed to specify a second dorsalizing organizer, the Spemann organizer, through Vg1 signaling[130]. A recent examination of Spemann organizer induction suggests a role for combinatorial signaling involving a TGF-β pathway and a pathway associated with the vertebrate homologs of Wingless (Wnt family members). In this study, the Wnt family signal transducers β-catenin and TCF formed complexes with Smad4, and these complexes synergistically activated the expression of organizer-specific genes[131]. Interestingly, combinatorial signaling by Wnts and the TGF-β family member cVg1 may also be required for primitive streak induction in the chick[132].

Complementing the dorsalizing activity of the two organizers, BMPs promote ventral mesoderm formation and are thought to form a dorsal–ventral morphogen gradient across the embryo. Recent antibody staining has revealed differential BMP4 localization in the pregastrula embryo, with highest levels at the ventral pole and no recognizable signals in the dorsal region[133]. Addition of Smad1 and Smad5 to early embryos also causes ventralization, suggesting that BMP4 is signaling through Smad1 and Smad5 to specify ventral cells[134,135]. Furthermore, removal of BMP activity from the ventral side of the embryo dorsalizes the entire embryo, as shown by the presence of ectopic neural tissue and the loss of epidermis[136,137,138]. BMPs therefore have a dual role in dorsal–ventral patterning; they promote the formation of ventral mesoderm and antagonize the formation of dorsal tissues such as the nervous system.

Dorsalization of the *Xenopus* blastula is accomplished, in part, through the activity of antagonists to ventralizing factors, such as BMP4. Noggin, Follistatin, and Chordin[139] play important roles in limiting the ability of BMP4 to ventralize the mesoderm and ectoderm. In addition, the TGF-β family members Activin and Vg1 actively induce dorsal mesoderm formation[140]. After gastrulation, Activin/Vg1 activity also patterns the endoderm, and ectopic application produces a second dorsal axis on the ventral side of the embryo[141]. Nodal-related, another TGF-β family member, is also required for dorsal mesoderm induction and endoderm patterning. In *Xenopus*, *nodal-related* transcription is upregulated by Activin[142], but inhibiting Nodal-related activity does not alter Activin's dorsal mesoderm-inducing ability, indicating that Activin signals via multiple mechanisms during dorsal–ventral patterning[143]. Similarly, Activin and two Nodal homologs also act to pattern the mesendoderm of zebrafish[144,145].

Inhibition of Nodal-related genes in *Xenopus* and zebrafish leads to defects in anterior–posterior patterning, including anterior truncations and loss of anterior, head-specific markers[143,146,147]. Similarly, anterior–posterior axis defects have been noted in Nodal-deficient mice[118]. These studies suggest that Nodal-related genes in *Xenopus*, zebrafish, and mouse actively promote the formation of anterior structures such as the head. Conversely, ectopic expression studies in *Xenopus* have shown that BMP2, BMP4, and BMP7 signaling antagonizes head formation[148] and that antagonists of BMP signals such as Cerebrus are activated in response to Nodal-related signals[147]. Taken together, head formation in *Xenopus*, zebrafish, and mouse appears to be mediated by antagonists to posterior-ventralizing factors such as BMPs. Roles for TGF-β family members in proximal–distal axis formation and left–right asymmetry in *Xenopus* have not been identified.

### *Fly — Axis Formation*

The anterior–posterior axis of *Drosophila* is patterned by the activation of a well-characterized hierarchy of gene expression independent of TGF-β signaling. TGF-β signaling is central to dorsal–ventral patterning in the fly, largely through the actions of the Dpp/BMP subfamily member *dpp.* Dpp transcripts are uniformly expressed throughout the dorsal 40–50% of the blastoderm embryo (Fig. 7A). However, post-translational activity involving antagonists and cooperation with other TGF-β family members generates a *dpp* activity gradient with the highest activity in the dorsal-most regions. This Dpp activity gradient complements a maternally

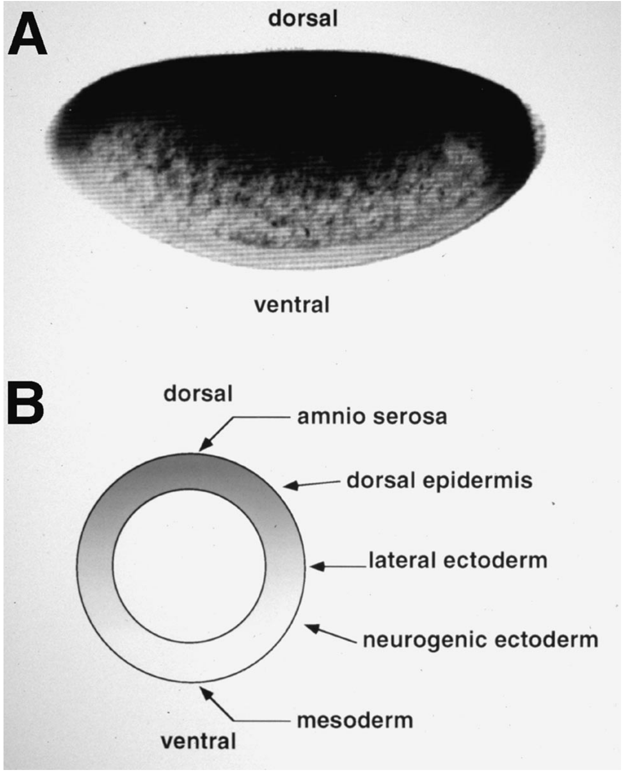

**FIGURE 7.** *dpp* expression and function in *Drosophila* embryonic dorsal–ventral axis formation. (A) A cellular blastoderm stage *Drosophila* embryo oriented anterior to the left and dorsal toward the top. The embryo is stained for *dpp* mRNA. Note that *dpp* expression is confined to the dorsal 50% of the embryo. (B) Schematic cross section of a cellular blastoderm embryo showing the eventual fate of cells from different regions along the dorsal–ventral axis. Within the cross section, a gradient of *dpp* activity is depicted. The dark area at the dorsal side indicates the highest level of *dpp* activity. The highest level of *dpp* activity induces dorsal cells to become the amnioserosa. Moving ventrally, the gradient lightens up until no *dpp* activity is detected in cells that eventually become the neurogenic ectoderm or mesoderm.

specified gradient of Dorsal activity[149,150]. The Dorsal protein specifies ventral cell fates, and Dpp signaling has been shown to limit the activity of Dorsal-mediated ventralization, consistent with Dpp's role as a dorsalizing factor[151]. The Dpp antagonist Short gastrulation (Sog) is critical to maintaining the slope of the Dpp gradient[48], particularly in the lateral ectoderm (Fig. 7B). In the lateral ectoderm, Dpp signaling promotes epidermal differentiation of cells whose default state is neural tissue. By antagonizing Dpp signaling in the lateral ectoderm, Sog promotes the formation of neural tissue. Interestingly, recent investigations have demonstrated that Sog can positively promote some aspects of Dpp gradient formation. Sog is required for peak levels of Dpp activity, and removal of Sog decreases the formation of the dorsal-most embryonic tissue[152,153].

A second Dpp/BMP subfamily member, *screw*, is also required for dorsal–ventral patterning[154]. Studies in *screw* mutant embryos using antibodies to phosphorylated Mad (pMad), the activated form of the Mad signal transducer, revealed that both *screw* and *dpp* are required to achieve wild-type pMad levels in the dorsal-most regions of the embryo[155]. Thus the proper specification of dorsal and lateral cell types along the dorsal–ventral axis is dependent on positive activity by Dpp and Screw signaling and antagonism of Dpp activity by Sog. To date, there is no strong evidence of left–right asymmetry in fly embryos. The proximal–distal axis is discussed in the section on limb formation.

### *Frog and Fly — Dorsal–Ventral Axis Inversion*

The observation that *dpp* specifies dorsal cell fates in *Drosophila* is in contrast to the ability of its vertebrate homolog (BMP4) to induce ventral cell fates in *Xenopus*. This apparent inconsistency has prompted a number of transgenic studies. For example, experiments have shown that Noggin, a dorsalizing factor in *Xenopus*, promotes ventral cell fates when expressed in *Drosophila,* specifically by antagonizing *dpp*[156]. Alternatively, human BMP4 rescues dorsal–ventral patterning defects in *dpp* mutant flies by promoting dorsal cell fates[4]. These results suggest that the molecular interactions utilized by proteins specifying dorsal–ventral axis formation have been conserved from invertebrates to vertebrates. Classical embryologists have long noted the inverse location of the central nervous system in the embryos of flies (ventral) and vertebrates (dorsal). Clearly, differences in the dorsal–ventral inducing activities of Dpp in *Drosophila* and BMP4 in *Xenopus* are associated with the fact that vertebrate dorsal–ventral axis formation is inverted with respect to that of flies.

### *Worm — Axis Formation*

In *C. elegans*, *unc-129* encodes a highly divergent Dpp/BMP subfamily member that is differentially expressed in the dorsal body wall musculature and in specific motor neurons[157,158]. Even though TGF-β signaling is not believed to play any major roles in axial patterning in nematodes, Unc-129 is thought to provide some dorsal–ventral information during neural development. During development of the nervous system, ventral nerve cord motor neurons project growth cones along the dorsal–ventral axis to the dorsal nerve cord[157,158]. Recently, a new gene influencing motor neuron migration, *unc-130*, was characterized. In *unc-130* mutants, ventral to dorsal neuron migration is absent, and *unc-129* expression expands to the ventral body wall musculature[158]. This suggests that Unc-129 provides dorsal–ventral positional cues, either directly or indirectly for neural growth cones, while Unc-130 antagonizes Unc-129 activity. Thus, antagonism of TGF-β family members appears to be a widespread mechanism for regulating TGF-β activity during development.

Genetic analyses have indicated that Unc-129 does not signal through Daf-4, the only Type II receptor identified in *C. elegans*. This unusual result suggests that Unc-129 utilizes a nonconventional mode of TGF-β signaling involving Type I receptors only[157]. Supporting this possibility, Daf-1, a nematode Type I receptor, can activate intracellular Smads in the absence of a Type II receptor[159]. Further characterization of TGF-β signaling during development in *C. elegans* may uncover previously unrecognized pathways.

## Tissue Patterning and Organogenesis

### *Mouse*

Signaling by TGF-β family members is required during the development of many vertebrate organ systems, including the nervous system[160], lungs[161], kidneys[162], heart[163], skin[136], and gonads[120]. For example, TGF-β–mediated epithelial–mesenchymal interactions are important during heart development, and Dpp/BMP proteins are required for proper skeletal patterning. TGF-β family members promote organ development by regulating numerous cellular processes such as branching morphogenesis, epithelial–mesenchymal transition, cell proliferation, and apoptosis.

In mice, branching morphogenesis is the process by which the tracheal branches of the lungs and the nephric ducts of the kidneys are formed. BMP4 is expressed at the tips of distal lung epithelial buds as well as in the surrounding mesenchyme. Overexpression of BMP4 in the lung buds reduces overall lung size[161], while overexpression of BMP antagonists or a dominantnegative BMP receptor in the lung buds reduces the number of fully differentiated cells[164,165]. These results suggest that BMP4 signaling represses cell proliferation while promoting the differentiation of lung buds into mature lung cells. During kidney development in mice, BMP signaling induces metanephric mesenchyme to differentiate into epithelium, and mice deficient for BMP7 display glomeruli-deficient kidneys[162].

### Chick

In the chick, the first subtype of mesoderm to arise after gastrulation is the prospective cardiac mesoderm. TGF-β/Activin subfamily signals from the hypoblast to the epiblast and Dpp/BMP subfamily signaling from the endoderm to the posterior mesoderm have both been implicated in the induction of cardiac mesoderm[115,166]. The subsequent formation of the myocardium (the heart muscle) is dependent on TGF-β, Activin, and BMP signals. Epicardium is deposited onto the myocardium by the proepicardial organ, a derivative of the primitive liver, and the epicardium will eventually fully surround the heart muscle. Coronary blood vessels and other supporting tissues are derived from the epicardium. Epithelial to mesenchymal transition is an essential part of epicardial differentiation, and epicardial epithelial to mesenchymal transition is regulated by TGF-β signaling[167].

TGF-β signaling is also required for epithelial to mesenchymal transition of endocardial cells (the cells that line the chambers of the heart), particularly those in the atrioventricular canal. Endocardial epithelial to mesenchymal transition is required for the formation of definitive heart structures such as valves and septa[168,169]. Interestingly, epithelial to mesenchymal transition in the endocardium and the epicardium seems to be under the control of different regulatory mechanisms. Specifically, endocardial epithelial to mesenchymal transition requires TGF-β2–induced expression of the transcription factor Slug[169]. In epicardial epithelial to mesenchymal transition, signaling by TGF-β1, TGF-β2, and TGF-β3 inhibits this process. Conversely, TGF-β antibodies that block signaling can stimulate epicardial epithelial to mesenchymal transition[167]. Thus, the role of TGF-β signaling during heart development is inductive in the case of the myocardium and endocardium but repressive during epicardial development.

### Fly

In *Drosophila*, Dpp signaling is required to pattern tissues derived from the ectoderm[170,171], mesoderm[3,172,173], and endoderm[174,175]. After Dpp patterns the dorsal and lateral ectoderm during axis formation, another round of Dpp signaling within the lateral ectoderm regulates the development of the tracheal system. Tracheal cells are first designated as a set of segmentally reiterated, bilaterally symmetrical placodes in the lateral ectoderm. Dpp signaling restricts the location along the dorsal–ventral axis within each segment in which tracheal placodes can form[171]. Once formed, cells within tracheal placodes migrate, form extended tubes, branch, and fuse across segment boundaries to form the interconnected tracheal system. A study employing dominant-negative and constitutively active forms of the Dpp receptor Thickveins indicates that a third round of ectodermal Dpp signaling promotes the extension of tracheal branches along the dorsal side of the embryo[176].

In the mesoderm, Dpp signaling from the dorsal ectoderm induces the specification of cardiac precursor cells. Dpp signaling specifies precardial cells by activating the homeobox gene *tinman*[3]. Tinman expression then induces dorsal mesodermal cells to differentiate into cardiac cells. Dpp signaling from the mesoderm patterns the underlying endoderm. The effect of this aspect of Dpp signaling is to induce a transient morphological constriction in the embryonic midgut and to indirectly induce the specification of midgut “copper cells” through activation of the homeotic gene *labial*[177,178]. A complex regulatory system insures proper Dpp signaling

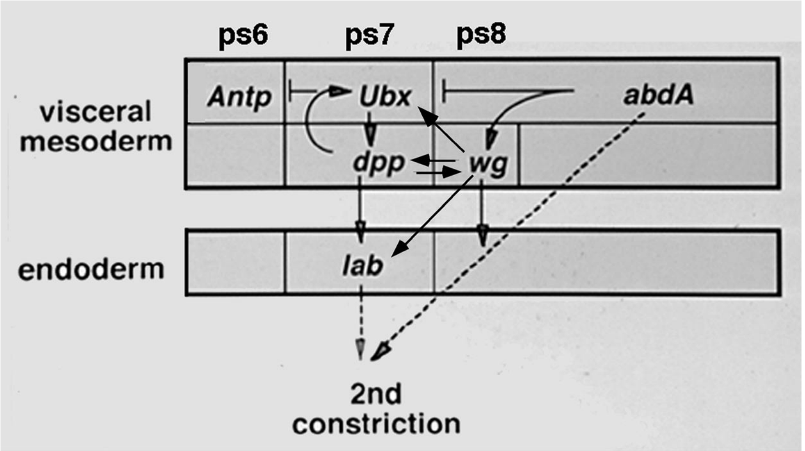

**FIGURE 8.** *dpp* activity in *Drosophila* embryonic midgut organogenesis. Schematic representation of genes expressed in two tissues (visceral mesoderm and endoderm) of the *Drosophila* embryonic midgut. A subset of the regulatory interactions necessary for the formation of the second midgut constriction and the eventual specification of "copper cells", in parasegments (ps) 6 through 8, are shown. Note that *dpp* (and *wg*) signals regulate the activity of several other genes in both tissues. This complex genetic network leads to a single morphological event.

within this region of the visceral mesoderm (Fig. 8). In the visceral mesoderm the transcription factor Ultrabithorax activates *dpp* expression. The maintenance of *dpp* expression depends upon Dpp autoregulation via Ultrabithorax. The maintenance of Dpp and Ultrabithorax expression also requires input from Wingless expressed in an adjacent region of the visceral mesoderm[19,28,179]. Furthermore, *wingless* expression depends upon feedback from Dpp. Both Wingless and Dpp signals are required to induce *labial* expression in the underlying endoderm[173,180]. Thus, paracrine regulatory loops involving both signaling pathways regulate the development of the embryonic midgut.

### Limb Formation

#### Chick

Axial patterning of the vertebrate limb requires cell–cell communication from two signaling centers, the zone of polarizing activity in the posterior–proximal limb bud mesoderm and the apical ectodermal ridge at the distal limb bud tip. The zone of polarizing activity sets up the overall anterior–posterior axis, and the apical ectodermal ridge patterns the limb's proximal–distal axis using a variety of signaling molecules, including TGF-β family members. Anterior–posterior patterning of the digits also involves TGF-β signaling.

In the chick, transplanting the zone of polarizing activity results in pattern duplications along the limb's anterior–posterior axis[181], demonstrating the zone of polarizing activity's role in anterior–posterior patterning. Molecular studies show that Sonic hedgehog, a signaling molecule expressed in the zone of polarizing activity, induces the expression of a number of genes, including BMP2[182]. A recent report suggests that cell fate along the anterior–posterior axis is specified by a gradient of BMP2 activity[183]. BMPs also play a role in proximal–distal axis formation. BMP2, BMP4, and BMP7 are expressed in the apical ectodermal ridge throughout limb development[184]. Unexpectedly, ectopic expression studies showed that signaling by these BMPs restricts limb outgrowth. The growth-inhibitory activity of these BMPs is neutralized during the early stages of limb development by transient expression of the BMP antagonist Gremlin[185,186].

Recent experiments in the chick demonstrate that the anterior–posterior identity of each digit depends upon BMP2, BMP4, or BMP7 signaling from interdigit regions[187]. Subsequently, BMP4 induces apoptosis of the interdigit regions, thereby removing tissue from between the chondritic cells from which the digits will develop[188,189]. Ectopic expression studies showed that the chondritic cells (cartilage precursors) of digits are specified by Activin and TGF-β2 signals. In these studies, Activin and TGF-β2 induce extra digits, while the Activin antagonist Follistatin reduces digit number[187,190]. Thus, TGF-β family members participate in patterning the overall anterior–posterior and proximal–distal axes of the vertebrate limb and contribute significantly to digit formation.

### Fly

In *Drosophila*, axial patterning and cell type specification in limb formation require multiple rounds of Dpp signaling. Here we describe the specification of leg primordia and several aspects of wing development where Dpp plays prominent roles.

Cells destined to become adult legs are specified during mid-stages of embryonic development. The earliest indicator that cells have committed to developing as legs is their expression of *distal-less*. *distal-less* is induced in cells of the lateral ectoderm by Wingless signaling. At the same time, Dpp signaling from the dorsal ectoderm acts to restrict the number of cells dedicated to becoming limbs by repressing *distal-less* expression in this region. Once their fate is determined, primordial limb cells migrate dorsally and invaginate to form imaginal disks, embryonic structures that give rise to adult limbs following metamorphosis[191].

Cells destined to become adult wings are also specified during mid-stages of embryonic development. Once a small group of embryonic cells has committed to forming wings, subsets of cells are segregated from each other by an unknown mechanism. Throughout the remainder of wing development (notwithstanding significant cell proliferation, migration, and cell fate specification), cells that will occupy anterior positions and cells that will occupy posterior regions of the adult wing never mix. The dividing line between anterior and posterior cells is called the anterior–posterior compartment boundary.

Expression of the transcriptional activator/repressor *engrailed* exclusively in cells of the posterior compartment leads to the activation of the secreted signaling molecule Hedgehog. Hedgehog is capable of migrating across the anterior–posterior compartment boundary. Hedgehog molecules that cross the boundary activate *dpp* expression in a narrow stripe just anterior to the anterior–posterior boundary. *dpp* expression is repressed throughout the posterior compartment by *engrailed* activity. *dpp* expression is restricted to the anterior–posterior compartment boundary by repression elsewhere in the anterior compartment mediated by the *patched* signaling protein[23,24]. Genetic analyses have shown that *dpp* activity patterns the entire wing, and molecular studies have shown that distinct sets of Dpp-responsive genes are activated at discrete distances from the stripe of *dpp* expression[63,192,193,194]. These studies led to a model in which Dpp diffusion results in a gradient of Dpp activity capable of stimulating differential gene expression throughout the developing wing (Fig. 9). The postulated gradient of *dpp* activity was eventually observed directly via the formation of a gradient of Mad nuclear localization and with fluorescently tagged Dpp.

This Dpp gradient is influenced by the activity of the TGF-β family member Glass bottom boat and then superimposed on cell-to-cell variation in Dpp receptor expression to achieve an appropriate gradient of anterior–posterior information[56,63,68,194]. In addition to anterior–posterior patterning, Dpp signaling also induces the formation of wing veins[195,196]. *dpp* is expressed in the pupal wing veins and acts to define both longitudinal and cross veins, while the Dpp antagonist *sog* is expressed in the intervein tissue[47]. *glass bottom boat* mutations result in vein abnormalities, suggesting that it also has a role in vein specification[197].

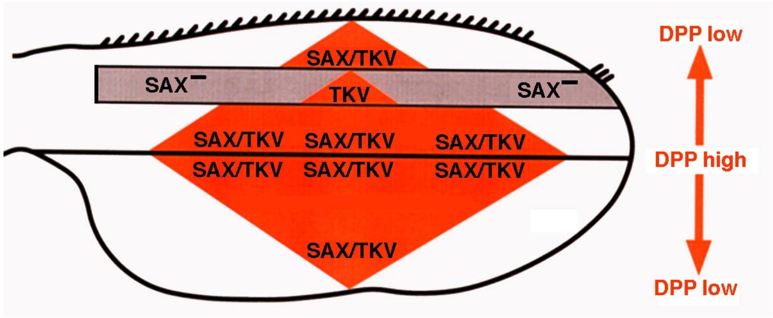

**FIGURE 9.** *dpp* activity in *Drosophila* adult wing anterior–posterior axis formation. Schematic representation of an adult wing oriented anterior toward the top. Within the wing, a gradient of Dpp activity is depicted as two triangles. The highest level of Dpp activity is found at the base of each triangle (along the anterior–posterior midline of the wing where Dpp is expressed). Dpp activity falls off as the distance from the midline increases. Dpp activity is at a minimum at the triangles' apex (the anterior and posterior wing margins). Superimposed upon the Dpp gradient in the anterior portion of the wing is a region containing cells with homozygous mutations in the TGF-β Type I receptor Saxophone (Sax-). Sax mutations effectively reduce Dpp signaling. This effect of Sax mutations on Dpp creates a second region of minimum Dpp activity posterior to the normal minimum. This second minimum is depicted as a triangle within the Sax- region. This second minimum leads cells in the Sax- region (cells normally exposed to a moderate level of Dpp activity) to adopt the fate of cells that normally see the minimum level of Dpp activity. This cell fate change is shown by the appearance of anterior wing margin hairs in cells posterior to their normal location.

#### *Worm*

Although nematodes lack classical appendages, male worms have tail rays. Tail ray patterning is under the control of a TGF-β pathway. This pathway involves the ligand Dbl1, the receptors Daf4 and Sma6, and the Smads Sma2, Sma3, and Sma4[81,198,199,200]. Mutations in each of these genes result in tail ray defects[198,199]. Interestingly, *dbl1* mutations result in reduced body size, while increased body size is seen when Dbl1 is overexpressed, suggesting that body size is a dosage-dependent response to TGF-β signaling[200].

### TUMOR SUPPRESSOR ACTIVITIES

TGF-β signaling induces embryonic cells to stop proliferating and to start differentiating in a wide variety of organisms. This suggests that TGF-β signals can influence the cell cycle during development. Within adult tissues, TGF-β signaling maintains homeostasis in a number of tissues. In adults, the loss of sensitivity to TGF-β signals (usually through signal transduction pathway mutations) can lead cells to overproliferate in these tissues. Thus, in adults, TGF-β pathways can have tumor suppressor activities. Alternatively, TGF-β signaling promotes cell proliferation in some developmental systems. In some adult tissues, TGF-β signaling can increase the invasiveness of some types of tumors. Thus, depending upon the circumstances, TGF-β signaling can have stimulative or inhibitory effects on the cell cycle. Here we examine the effects of TGF-β signaling on the cell cycle and the involvement of TGF-β signaling pathway mutations in colon, pancreatic, and breast cancers.

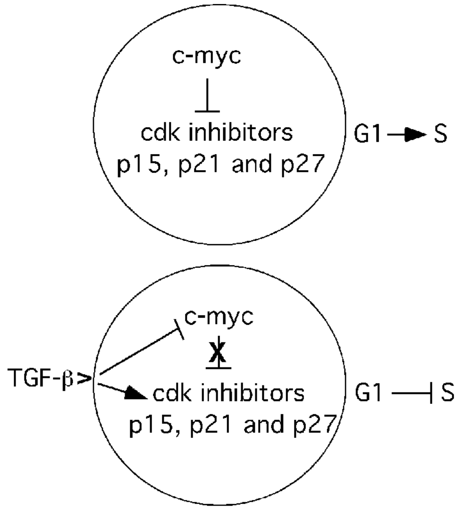

**FIGURE 10.** TGF-β induces cell cycle arrest in epithelial cells and keratinocytes. Top panel: During normal cell cycle progression, c-Myc represses the transcription of the cyclin-dependent kinase (cdk) inhibitors p15, p21, and p27. This repression permits the cell's transition from the first growth phase (G1) to the DNA synthesis phase (S). Bottom panel: TGF-β arrests the cell cycle via synergistic mechanisms. TGF-β signals repress the transcription of c-Myc, thereby preventing c-Myc repression of cdk inhibitor expression. At the same time, TGF-β signals actively stimulate the transcription of the cdk inhibitors. These synergistic activities prevent the cell's transition from the first growth phase to the DNA synthesis phase.

### Cell Cycle Control

All eukaryotic cells progress through a cycle of growth, DNA synthesis, growth, and mitosis in order to reproduce. Regulation of this cell cycle is often accomplished by controlling the ability of a cell to proceed from the first growth phase (G1) to the DNA synthesis phase (S). The transition from G1 to S phase is dependent on two types of proteins: cyclins and cyclin-dependent kinases (cdks). Through an elaborate pathway, cyclins and cdks influence the release of the transcription factor E2F from a complex with the Retinoblastoma protein. E2F then initiates the transcription of genes required for DNA synthesis. Signaling by TGF-β family members can block a cell's transition from G1 to S phase, a process known as growth arrest, by downregulating the activity of cdks.

TGF-β signaling influences cdk activity by modulating the transcriptional activity of cdk inhibitors. Signals from several TGF-β/Activin subfamily members increase the activity of the cdk inhibitors p15, p21, and p27 (Fig. 10). One mechanism by which TGF-β signaling stimulates cdk inhibitor expression is via the regulation of the transcription factor c-Myc. c-Myc is an important cell cycle regulator in epithelial cells and keratinocytes. In these cells, c-Myc is a transcriptional repressor of the cdk inhibitors p15, p21, and p27, and therefore c-Myc activity permits cell cycle progression[201,202,203,204]. Adding TGF-β to these cells in culture results in the repression of c-Myc expression and effectively prevents c-Myc repression of cdk inhibitor expression. Alternatively, the ectopic expression of c-Myc desensitizes these cells to TGF-β–mediated activation of cdk inhibitors[203,204,205]. Recent reporter gene experiments have identified a TGF-β–Inhibitory Element in the c-Myc promoter. In response to TGF-β signals, Smad complexes bind this element and dramatically reduce c-Myc expression[205]. Thus, TGF-β–induced c-Myc repression leads to cdk inhibitor transcription and cell cycle arrest. Mutations in c-Myc's TGF-β–Inhibitory Element result in a loss of sensitivity to TGF-β–mediated growth arrest.

Isoforms of TGF-β can also regulate cell proliferation by a direct transcriptional mechanism. For example, TGF-β1 signaling stimulates the transcription of a p21 reporter gene in hepatoma cells[206] and keratinocytes[207]. Subsequently, a TGF-β response element was identified in the p21 promoter. Gel shift assays indicate that Smad3/Smad4 complexes, in association with the transcription factor Sp1, specifically bind to this element[207].

Members of the TGF-β/Activin subfamily can also regulate cell proliferation through translational control of cdk mRNAs. In mink lung epithelial cells, addition of TGF-β decreases the production of cdk4 protein but does not alter the level of cdk4 mRNA[208,209]. Subsequent studies showed that, in response to TGF-β signaling, the 5' untranslated region of cdk4 mRNA is bound by p53, effectively decreasing its translational efficiency[209].

Dpp/BMP subfamily members have also been implicated in cdk inhibitor activation. BMP4 signaling increases p21 levels in neuronal and dental epithelial cells. However, activation of p21 in these cells leads to apoptosis and not growth arrest[210,211]. Alternatively, BMP2 signaling directly activates p21 in B-cells and in breast cancer cells, inhibiting cell proliferation[212,213].

Dpp signaling regulates cell cycle progression in the developing *Drosophila* eye. In the eye, a moving wave of cellular differentiation (one row at a time, groups of pluripotent cells become neurons, photoreceptors, and support cells simultaneously) is known as the morphogenetic furrow. Dpp signals emanate from differentiated cells within the morphogenetic furrow. Dpp then diffuses ahead of the differentiation wave and temporarily induces growth arrest in undifferentiated cells. This appears to occur via an unidentified cdk inhibitor. The activity of Dpp effectively synchronizes the cell cycle in the undifferentiated cells prior to their entering the morphogenetic furrow[214,215]. Thus, cdk inhibitor activation appears to be a cell cycle control mechanism used by TGF-β/Activin and Dpp/BMP subfamily members.

### Colon, Breast, and Pancreatic Cancers

TGF-β signaling can induce growth arrest in cells from a variety of tissues, implying that TGF-β signal transduction pathway components have tumor suppressor activities. Homozygous mutations in signal transduction pathway genes and in TGF-β target genes, such as c-Myc, have been identified in a number of carcinomas (Table 3). Mutations in these genes result in insensitivity to TGF-β–mediated growth control and unregulated cell proliferation.

Microsatellite instability is common in a number of tumors and results from defects in the DNA mismatch repair machinery[216]. Microsatellites are short, highly repetitive DNA sequences that are often subject to replication errors. The most common replication errors result in increases or decreases in the number of repeat units within a particular microsatellite. In cells defective for mismatch repair, such replication errors remain uncorrected, leading to microsatellite instability. Microsatellite instability is present in 15% of colorectal carcinomas[217], and up to 90% of microsatellite instability–positive colorectal carcinomas manifest mutations in the TGF-β type II receptor[217,218]. This high frequency of TGF-β type II receptor mutations is most likely due to the presence of a 10-nucleotide run of adenines and a G/T dinucleotide repeat in the protein coding region[219]. Expansion or contraction of these microsatellite sequences typically results in a frameshift mutation that produces a truncated receptor unable to transduce TGF-β signals. Interestingly, in a study of microsatellite instability–positive breast tumors, none showed mutations in the TGF-β type II receptor[220].

Colorectal cancers without microsatellite instability can also contain TGF-β signal transduction pathway mutations. In a recent study of 44 colorectal cancer cell lines, 32 did not show microsatellite instability or TGF-β type II receptor mutations[221]. On the other hand, 14 of these microsatellite instability–negative lines demonstrated a loss of Smad4 protein expression.

**TABLE 3**

**Survey of TGF-β Pathway Defects in Human Cancers**

| Tissue Type | Gene | Alteration | Incidence (%) | Reference |
|---|---|---|---|---|
| Prostate | TBRII | Loss of expression | 25 | 243 |
| | TBRII | Loss of expression | 13 | 244 |
| | TBRI | Loss of expression | 25 | 243,244 |
| Gastric | unknown | Impaired signaling | 40 | 245 |
| | TBRII | Deletions, transitions, and transversions | 10 | 246 |
| | TBRI | Downregulation | 13 | 247 |
| Hepatocellular | TBRII | Downregulation | 50 | 248 |
| | Smad2 | Transition | 3 | 249 |
| | Smad4 | Transition | 6 | 249 |
| Primary biliary tract | Smad4 | Point mutation | 16 | 250 |
| Common bile duct | Smad4 | Inactivation | 50 | 250 |
| Lung adenocarcinoma | TBRII | Downregulation | 62 | 251 |
| Cervical | TBRII | Nonsense mutation | 6 | 251 |
| Pancreatic | TBRII | Frameshifts and deletions | 4 | 230 |
| | Smad4 | Homozygous deletion | 30 | 2 |
| | Smad4 | Inactivation | 20 | 2 |
| Colon | TBRII | Inactivation ($MIS^+$) | 90 | 218 |
| | Smad4 | Inactivation (non-MIS) | 42 | 221 |

None of the 12 microsatellite instability–positive lines displayed loss of Smad4, suggesting that loss of Smad4 is common in microsatellite instability–negative colorectal carcinomas. Thus, colorectal cancers characterized by microsatellite instability are likely to contain TGF-β type II receptor mutations, while a significant portion of those without microsatellite instability contain mutations in Smad4. In either event, colorectal cells without a TGF-β type II receptor or Smad4 cannot respond to TGF-β–mediated growth control.

Breast tumors were the first cancer cells to be identified with a loss of TGF-β–mediated growth inhibition[222]. To date, four mechanisms for TGF-β insensitivity have been characterized in breast cancers: loss of TGF-β Type II receptor function, loss of Smad4 function, mutations in the TGF-β target c-Myc, and overexpression of the TGF-β antagonist murine double minute 2 (MDM2)[205,223,224,225]. TGF-β Type II receptor mutations have been documented in a mouse model for breast cancer[226] and in a number of breast cancer cell lines[223,227]. Other breast cancer cell lines produce a TGF-β Type II receptor protein, but the receptor is unable to associate with the cell membrane[228]. There are also breast cancer cell lines with functional TGF-β Type II receptors that remain TGF-β insensitive due to mutations in Smad4[227]. When these cell lines were transfected with wild-type Smad4, TGF-β–induced growth arrest was restored[224].

Alterations in two cell cycle regulators, c-Myc and MDM2, can also confer TGF-β insensitivity in breast cancer cells. As mentioned above, mutations in the TGF-β–Inhibitory Element of the c-Myc gene abrogate the ability of TGF-β signaling to activate cdk inhibitor expression in breast cancer cells[205]. MDM2 negatively regulates p53, which is known to mediate TGF-β regulation of cdk4[209]. Several breast cancer lines overexpress MDM2, and these cells are insensitive to the growth-inhibitory signals of TGF-β[225]. Interestingly, MDM2 overexpression in lung epithelial cells does not affect TGF-β–mediated growth arrest, suggesting that MDM2 involvement in blocking the TGF-β growth arrest pathway is tissue specific[229].

Pancreatic tumors were the first cancers in which the tumor suppressor activity of Smad4 was identified, as indicated by its original name, DPC4 (deleted in pancreatic cancer, locus 4).

Loss of the chromosomal region containing Smad4 is implicated in approximately 90% of pancreatic cancers, and of these 50–80% have mutations in Smad4[2,230]. Subsequent studies have concluded that mutations in TGF-β signal transduction pathway components or target genes such as c-Myc are present in nearly all pancreatic cancers[230].

Loss of TGF-β signal transduction pathway components is by no means limited to colon, breast, and pancreatic cancers. At low frequencies, TGF-β pathway mutations are found in tumors from many tissues[231,232,233].

### FUTURE DIRECTIONS

Notwithstanding its documented versatility, the number of known cellular responses to TGF-β signaling is relatively small when compared to the amount of intercellular communication required during embryonic development and adult homeostasis. It is becoming clear that TGF-β signals are often used in combination with other signaling pathways to generate unique cellular responses[234]. At this time, very little information is available about how signaling pathways are integrated in any developmental or adult system. Considerable effort is now devoted to understanding combinatorial signaling, and this issue is likely to dominate the field of TGF-β signaling for some time.

### ACKNOWLEDGEMENTS

We thank Deb Hursh, Karen Lyons, Russell Nicholls, Matt Singer, and Vern Twombly for assistance with figures. We thank Jessica Johnson for help in manuscript preparation. Studies of TGF-β signaling in the Newfeld lab are supported by a Basil O'Connor Starter Scholar Research Award from the March of Dimes.
